# Pharmacokinetics of Geraniol and Its Metabolites in Mice After Oral Administration

**DOI:** 10.1002/fsn3.4653

**Published:** 2024-12-09

**Authors:** Yoshiaki Moriki, Ryo Mitsugi, Tomoyoshi Kayou, Jumpei Horikoshi, Yoshimasa Yamaguchi, Shuichi Shibuya, Takahiko Shimizu

**Affiliations:** ^1^ R&D Center, Zenyaku Kogyo Co. Ltd. Hachioji‐shi Tokyo Japan; ^2^ Zenyaku Kogyo Co. Ltd. Bunkyo‐ku Tokyo Japan; ^3^ PlasMEDi Inc. Ariake Koto‐ku Tokyo Japan; ^4^ Aging Stress Response Research Project Team, National Center for Geriatrics and Gerontology Obu‐city Aichi Japan; ^5^ Department of Regenerative Medicine Faculty of Pharmacy, Sanyo‐Onoda City University Yamaguchi Japan; ^6^ Department of Food and Reproductive Function Advanced Research Juntendo University Graduate School of Medicine Bunkyo‐ku Tokyo Japan

**Keywords:** conjugated metabolites, geranic acid, geraniol, pharmacokinetics

## Abstract

Geraniol is an acyclic monoterpene alcohol that is extracted from the essential oils of aromatic plants. Geraniol has several biological activities such as anti‐cancer, anti‐inflammatory, antioxidant, and neuroprotective effects. However, the pharmacokinetics of geraniol and its metabolites after oral administration remain unknown in mice. To investigate the pharmacokinetics, the blood concentrations were measured in C57BL/6J mice by LC‐MS/MS after oral administration of geraniol at a dose of 200 mg/kg. The *C*_max_ for blood levels of geraniol was only 0.05 ± 0.01 μg/mL at 1 h after administration. In contrast, geranic acid, one of the geraniol metabolites, rapidly reached a peak level that was markedly higher than that of geraniol. Furthermore, the glucuronide conjugate of geraniol was detected at a higher level than geraniol. These results indicate that geraniol is rapidly converted to geranic acid or glucuronide conjugate after oral administration. Moreover, geraniol was detected in the liver and the brain, whereas 8‐hydroxygeraniol was not detected in any tissues. In contrast, geranic acid was detected in several tissues in the order of kidney > liver = lung > brain. Therefore, the metabolites of geraniol are present in the blood and tissues of mice treated with geraniol, and various pharmacological effects of geraniol may be caused by its metabolites.

## Graphical abstract

Geraniol is an acyclic monoterpene alcohol that is extracted from the essential oils of aromatic plants. Geraniol is rapidly converted to geranic acid or glucuronide conjugate after oral administration. The metabolites of geraniol are present in the blood and tissues of mice treated with geraniol, and various pharmacological effects of geraniol may be caused by its metabolites.

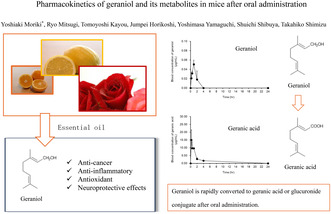

## 1 Introduction

Geraniol (3,7‐dimethylocta‐trans‐2,6‐dien‐1‐ol) is an acyclic monoterpene whose presence is abundant in essential oils extracted from aromatic plants such as ginger, lemon, orange, and rose (Lapczynski et al. 2008). Geraniol has been shown to exert a wide spectrum of pharmacological activities, namely anti‐inflammatory (Marcuzzi, Crovella, and Pontillo 2011; Wang et al. 2016), anti‐microbial (Thapa et al. 2012), anti‐oxidant (Khan et al. 2013; El‐Emam et al. 2020), and neuroprotective (Rekha, Selvakumar, Santha, et al. 2013; Rekha, Selvakumar, Sethupathy, et al. 2013; Rekha and Selvakumar 2014) effects. For example, oral administration of geraniol prevents colitis‐associated dysbiosis and decreases the inflammatory systemic profile of the colitis mouse model (De Fazio et al. 2016). Moreover, the anti‐tumor effects of geraniol have been evaluated in a broad range of cancers, including kidney, liver, lung, and pancreatic cancers (Cho et al. 2016). Oral administration of geraniol decreases tumor weight and volume in the non‐small‐cell lung cancer cell lines in A549‐implanted nude mice (Galle et al. 2014). Recently, it has been reported that geraniol blocks vascular endothelial growth factor (VEGF)/VEGFR‐2 signal transduction, and oral administration of geraniol inhibits the vascularization of CT26 tumors in the dorsal skinfold chambers of BALB/c mice (Wittig et al. 2015).

Previously, the pharmacokinetic profiles for two formulations of geraniol have been compared and analyzed in rats (Pavan et al. 2018). The bioavailability of the emulsified formulation using anhydrous glycerol is higher than that of the vegetable fiber‐absorbed formulation in rats, suggesting that the absorption is limited by its dissolution because geraniol has poor solubility and a relatively high n‐octanol partition coefficient (Turina et al. 2006). Additionally, geraniol can permeate directly from the blood to the brain following oral administration in the emulsified form. Recently, it has been shown that oral administration of an emulsified formulation using the amphiphilic polymer chitosan‐oleate induces a significant increase in both the bioavailability and level of geraniol in the brain (Bonferoni et al. 2019).

**FIGURE 1:**
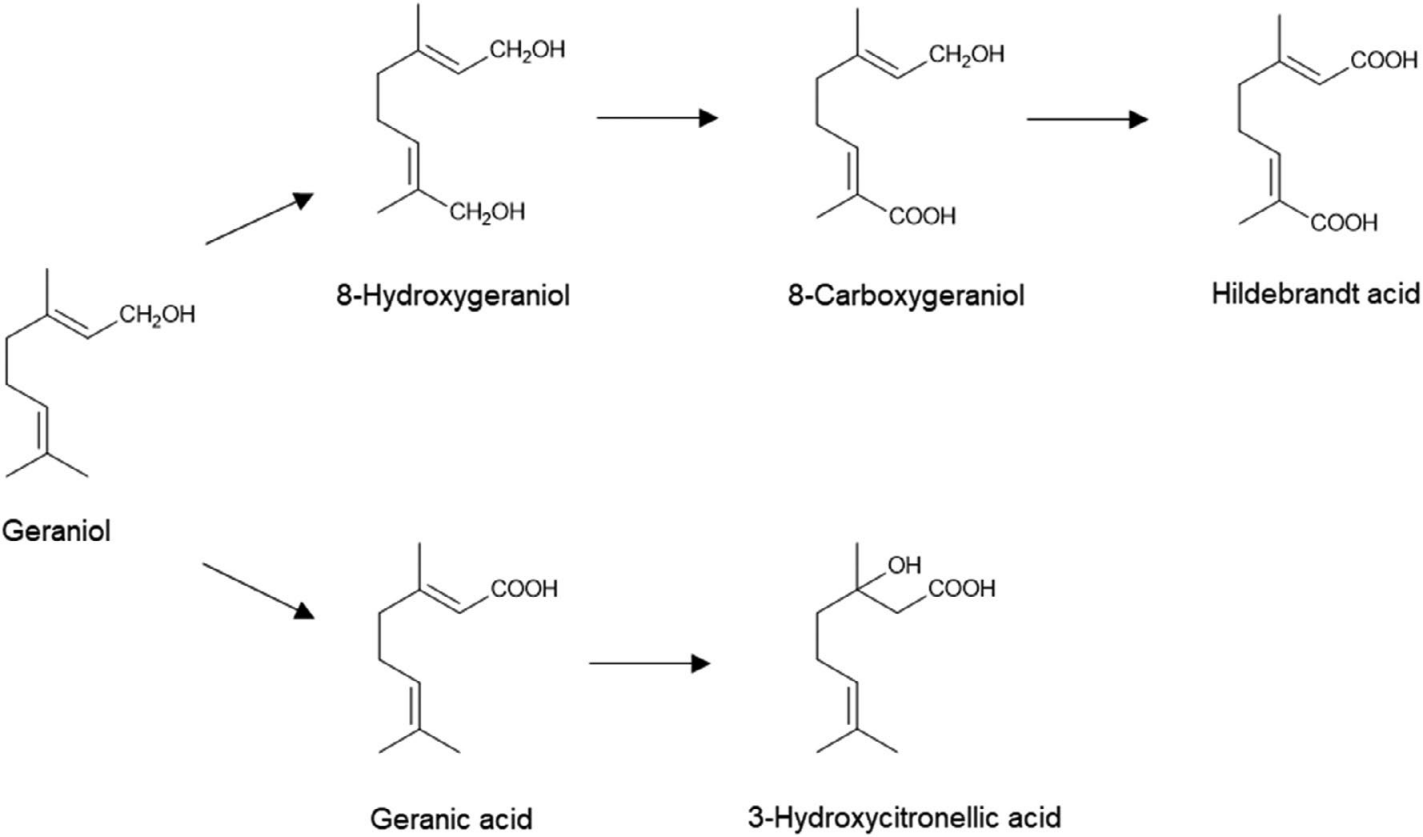
Metabolic pathway of geraniol (Chadha and Madyastha 1984).

The profile of geraniol metabolites has been investigated in insects (Takechi and Miyazawa 2006), rats (Chadha and Madyastha 1984), and humans (Jäger et al. 2020). Geranic acid, 3‐hydroxycitronellic acid, 8‐hydroxygeraniol, 8‐carboxygeraniol, and Hildebrandt acid have been detected in the urine of rats after oral administration of geraniol, and the metabolic pathway of geraniol has been proposed (Figure 1) (Chadha and Madyastha 1984). Recently, Jäger et al. (2020) reported that 3‐hydroxycitronelic acid, 8‐carboxygeraniol, geranic acid, and Hildebrandt acid, but not 8‐hydroxygeraniol, were detected in human urine. However, the profile of geraniol metabolites after oral administration in mice has not been fully verified.

To clarify several pharmacological effects of geraniol, it is necessary to elucidate the pharmacokinetics of geraniol and its metabolites. In the present study, we investigated the pharmacokinetics of geraniol and its metabolites after oral administration of geraniol in mice.

## 2 Materials and Methods

### 2.1 Animals

Animal care and all experimental procedures were performed with the approval of the Institutional Animal Care and Use Committee of Zenyaku Kogyo Co. Ltd, in terms of the three Rs (Replacement/Reduction/Refinement). Male C57BL/6J mice (7 weeks old, 20–27 g) were purchased from the Jackson Laboratory Japan Inc. (Kanagawa, Japan). The mice were housed under a 12‐h light/dark cycle at a constant temperature of 23°C ± 3°C and relative humidity of 50% ± 10% and had free access to feed and water. The animals had been acclimatized for 1 week before the start of the experiment.

### 2.2 Materials

Geraniol and corn oil were purchased from FUJIFILM Wako Pure Chemical Corporation (Osaka, Japan). Geranic acid, 8‐hydroxygeraniol, and geraniol‐D6 were obtained from Thermo Fisher Scientific (Waltham, MA, USA), Santa Cruz Biotechnology Inc. (Dallas, TX, USA), and Toronto Research Chemicals (Toronto, Canada), respectively. Geraniol‐D6 is a deuterium‐labeled analogue of geraniol. Geraniol‐D6 was used as an internal standard to prevent loss of accuracy due to matrix effects. Beta‐glucuronidase from bovine liver was purchased from Sigma‐Aldrich (St. Louis, MO, USA). All chemicals were of analytical grade.

### 2.3 Administration of Geraniol to Mice: Blood and Tissue Sampling

A total of 18 mice were used for study in which geraniol was mixed with corn oil and orally administered to the mice at a dose of 200 mg/kg (11.35 mL/kg) by gavage. For blood and tissue collection, *n* = 3 at each time point. The blood and tissue samples were collected at 0.25, 0.5, 1, 2, 4, and 24 h after oral administration under isoflurane anesthesia. The abdominal cavity of the anesthetized mouse was opened, and blood was collected from the posterior vena cava. After euthanasia, tissue samples were collected. The tissue samples were rinsed with phosphate‐buffed saline to remove the blood. The blood (100 μL) and tissue samples were stored at −30°C until the analysis. The rest of the blood samples were put in tubes with 2 K EDTA (CJ‐2DK, Terumo co.jp, Japan), followed by centrifuging at approximately 3000 rpm for 10 min. The resulting plasma layers were separated and stored at −30°C until the analysis. Before conducting these experiments, we verified that the concentrations of geraniol and geranic acid in the plasma samples, the blood samples, and the liver tissue samples were stable between immediately after preparation and after storage at −30°C for 3 days (data not shown). We also verified that geraniol, geranic acid, and 8‐hydroxygeraniol in mouse liver microsome samples were stable at −30°C for 3 days (data not shown). We believe that geraniol, geranic acid, and 8‐hydroxygeraniol are stable during the experimental period.

### 2.4 Blood Samples Preparation

The blood samples (100 μL), internal standard solution (100 μL, geraniol‐D6, 40 μg/mL acetonitrile), and acetonitrile (200 μL) were mixed and centrifuged at 4°C (10,000 rpm, 10 min), and the supernatant (10 μL) was subjected to LC‐MS/MS (Alliance 2695 Separations Module and Quattro micro API, Waters Co., MA, USA).

### 2.5 Tissue Samples Preparation

Five hundred milligrams of tissue samples was placed in microcentrifuge tubes and homogenized with distilled water (500 μL) using a homogenizer (Digital Homogenizer, Iuchi Seieido Co. Ltd., Japan). The biological samples (100 μL), internal standard solution (100 μL, geraniol‐D6, 40 μg/mL acetonitrile), and acetonitrile (200 μL) were mixed and centrifuged at 4°C (10,000 rpm, 10 min), and the supernatant (10 μL) was subjected to LC‐MS/MS.

### 2.6 Hydrolysis by Beta‐Glucuronidase

The plasma samples (100 μL) were transferred into 1.5‐mL tubes and mixed for 1 min with 100 μL of beta‐glucuronidase solution, which was prepared in 0.2 M phosphate buffer (pH 6.8) at a concentration of 40,500 units/mL. Then, the samples were incubated at 37°C for 1 h. After incubation, 400 μL of acetonitrile was added and mixed to stop the reaction. After centrifugation for 10 min at 4°C and 10,000 rpm, the supernatant was analyzed by LC‐MS/MS.

### 2.7 Quantitative Analysis of Geraniol, Geranic Acid, and 8‐Hydroxygeraniol

The concentrations of geraniol, geranic acid, and 8‐hydroxygeraniol in all samples were measured by LC‐MS/MS. The LC separation was performed using a Sunfire C_18_ column (3.5 μm, 2.1 × 150 mm (Waters Co.)) and Sunfire C_18_ guard column (3.5 μm, 2.1 × 10 mm (Waters Co.)). The mobile phase consisted of acetonitrile and 0.1% formic acid (50:50, v/v) at a flow rate of 0.2 mL/min. The injection volume was 10 μL, and the system was operated at 40°C. Geraniol, geranic acid, 8‐hydroxygeraniol, and geraniol‐D6 were eluted at a peak retention time of 9.4, 8.9, 2.9, and 9.2 min, respectively, with total run time of 12 min. MS was performed in the electrospray positive ionization mode. The MS conditions were set as follows: capillary voltage of 4 kV, source temperature of 110°C, cone gas flow of 50 L/h, and desolvation gas flow of 350 L/h. Quantitative analysis was performed in the multiple reaction monitoring (MRM) mode. The MRM transitions (Cone V, Collision eV) were m/z 137.1–80.9 (10, 10) for geraniol, m/z 169.2–123.0 (15, 10) for geranic acid, m/z 153.1–107.0 (15, 15) for 8‐hydroxygeraniol, and m/z 143.1–86.9 (10, 10) for geraniol‐D6. The ranges of the calibration curves for each compound from all tissue samples were obtained as follows: geraniol (blood), 0.016–10 μg/mL; geraniol (kidney, brain, liver), 0.08–20 μg/g; geraniol (lung), 0.16–20 μg/g; geranic acid (blood), 0.064–40 μg/mL; geranic acid (kidney, lung), 0.16–20 μg/g; geranic acid (brain), 0.16–20 μg/g; geranic acid (liver), 0.4–20 μg/g; 8‐hydroxygeraiol (blood), 0.08–10 μg/mL; 8‐hydroxygeraniol (kidney, lung), 0.08–20 μg/g; 8‐hydroxygeraniol (brain, liver), 0.16–20 μg/g. Concentrations below the lower limit of the calibration curve were set to 0 for geraniol, geranic acid, and 8‐hydroxylated geraniol samples.

### 2.8 Pharmacokinetic Analysis and Determination of Pharmacokinetic Parameters

The mean and standard error of concentration in blood and tissues at each time point were calculated using the values obtained from the three animals at each time point. In this study, values below the calibration curve were not excluded and were calculated as 0. The pharmacokinetic parameters in blood and several tissues were determined using a noncompartmental analysis method (NCA) using the sparse sampling mode with Phoenix WinNonlin Ver. 8.2 (Pharsight Corp., CA, USA). However, the standard error of the half‐life was calculated manually.

## 3 Results

### 3.1 Blood Concentration and Pharmacokinetic Parameters of Geraniol and Its Metabolites

**FIGURE 2:**
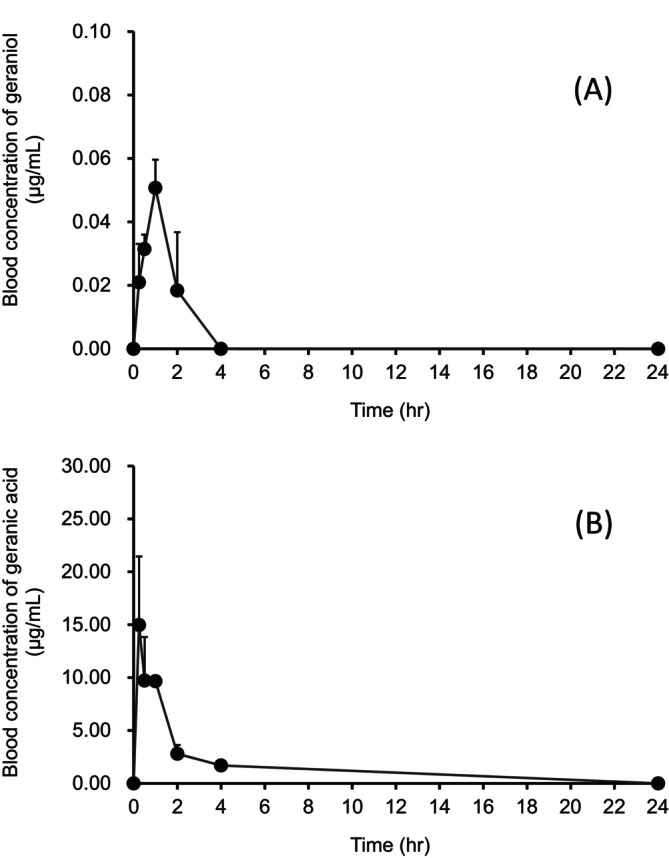
Blood concentration of geraniol (A) and geranic acid (B) following oral administration of geraniol. Data expressed as the mean ± SE (*n* = 3).

Geraniol reached a *C*_max_ of 0.05 ± 0.01 μg/mL at 1 h after oral administration in the blood (Figure 2, Table 1). The blood concentration of geraniol decreased rapidly to levels below the limit of quantification at 4 h after administration. Geranic acid was rapidly detected after administration of geraniol, and the *C*_max_ (14.97 ± 6.48 μg/mL) was approximately 300 times higher than that of geraniol. The *AUC* (37.49 ± 5.04 μg/mL × h) of geranic acid was approximately 470 times higher than that of geraniol. The calculated half‐life (*T*_1/2_) of geranic acid was 1.29 ± 0.33 h. However, 8‐hydroxygeraniol was not detected.

**TABLE 1:** Pharmacokinetic parameters of geraniol and geranic acid after oral administration of geraniol^a^.

	*C*_max_ (μg/mL)	*T*_max_ (h)	*AUC* (μg/mL × h)	*T*_1/2_ (h)
Geraniol	0.05 ± 0.01	1.00	0.08 ± 0.03	n.c.
Geranic acid	14.97 ± 6.48	0.25	37.49 ± 5.04	1.29 ± 0.33

*Note:* Data expressed as the mean ± SE.

Abbreviation: n.c., not calculated.

^a^ Parameters were calculated by analysis of the blood concentration versus time plots using the sparse sampling mode in the NCA module of the Phoenix WinNonlin Ver. 8.2.

### 3.2 Hydrolysis by Beta‐Glucuronidase

**FIGURE 3:**
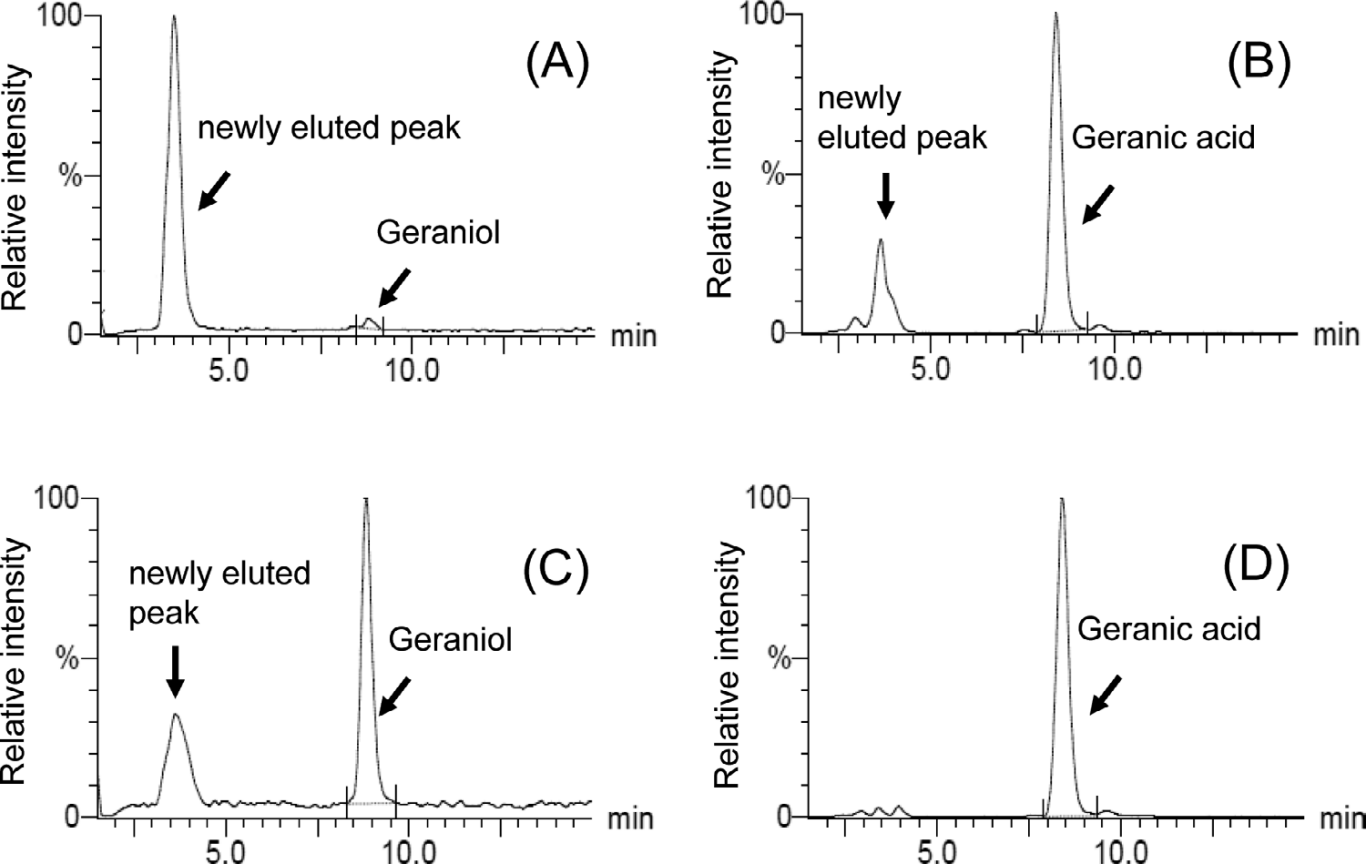
MRM chromatograms of geraniol and geranic acid in pooled plasma samples at 1 h after oral administration of geraniol. (A) MRM chromatogram of geraniol in a pooled plasma sample without beta‐glucuronidase, (B) MRM chromatogram of geranic acid in a pooled plasma sample without beta‐glucuronidase, (C) MRM chromatogram of geraniol in a pooled plasma sample with beta‐glucuronidase, and (D) MRM chromatogram of geranic acid in a pooled plasma sample with beta‐glucuronidase.

**FIGURE 4:**
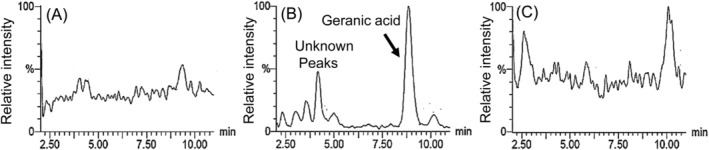
MRM chromatograms of geraniol (A), geranic acid (B), and 8‐hydroxygeraniol (C) in the brain at 1 h after oral administration of geraniol.

Figure 3 shows MRM chromatograms of geraniol and geranic acid in the plasma at 1 h after oral administration of geraniol. The pooled plasma samples with or without beta‐glucuronidases were analyzed to assess the effect of enzymatic hydrolysis. The levels of geraniol and geranic acid increased in incubation with beta‐glucuronidase compared with the control (without hydrolysis), whereas the levels of newly eluted peaks decreased. In contrast, 8‐hydroxygeraniol levels did not change compared with the control (data not shown). These results indicate that geraniol and geranic acid metabolized to the glucuronide conjugate. Moreover, we found unknown peaks in the MRM chromatograms of geranic acid, but not geraniol and 8‐hyroxygeraniol, in the brain (Figure 4). These results suggest that the unknown peak may be a glucuronide or sulfate conjugate of geranic acid metabolized in the brain.

### 3.3 Several Tissues Concentration and Pharmacokinetic Parameters of Geraniol and Its Metabolites

Geraniol was detected in the liver at 0.5, 1, 2, and 4 h and in the brain at 4 h after oral administration, although geraniol was only detected in the brain of one animal at a concentration of 0.106 μg/g (Table 2). Furthermore, 8‐hydroxygeraniol was not detected in any tissues. In contrast, geranic acid was rapidly and widely distributed in the kidney, lungs, liver, and brain. The level of geranic acid in the kidney was the highest, followed by the liver, lungs, and brain. The brain level of geranic acid was the lowest, suggesting that the transport of geranic acid into the brain was limited by the blood–brain barrier. The *C*_max_ for geraniol reached 0.21 ± 0.04 μg/g at 1 h after administration and then decreased in the liver (Table 3). In addition, the *AUC* of geranic acid was higher than that of geraniol in the liver, similar to the blood. Geraniol and geranic acid were not detected in any tissue samples at 24 h after administration.

**TABLE 2:** Concentrations of geraniol and geranic acid in several tissues after oral administration of geraniol.

		Concentration (μg/g)				
		0.25 h	0.5 h	1 h	2 h	4 h
Brain	Geraniol	n.d.	n.d.	n.d.	n.d.	0.106^a^
	Geranic acid	1.14 ± 0.62	0.86 ± 0.55	1.38 ± 0.29	0.10 ± 0.10	n.d.
Kidney	Geraniol	n.d.	n.d.	n.d.	n.d.	n.d.
	Geranic acid	7.25 ± 1.99	18.29 ± 11.81	86.57 ± 10.13	3.87 ± 2.28	1.46 ± 0.25
Liver	Geraniol	n.d.	0.07 ± 0.04	0.21 ± 0.04	0.19 ± 0.02	0.11 ± 0.06
	Geranic acid	2.24 ± 0.80	3.53 ± 1.70	6.31 ± 1.64	2.89 ± 0.64	1.52 ± 0.20
Lung	Geraniol	n.d.	n.d.	n.d.	n.d.	n.d.
	Geranic acid	4.18 ± 1.88	3.43 ± 0.52	8.53 ± 1.29	1.85 ± 0.66	1.45 ± 0.79

*Note:* Data expressed as the mean ± SE (*n* = 3).

Abbreviation: n.d., not detected.

^a^ This value was obtained from one animal. Geraniol was not detected in the other two animals.

**TABLE 3:** Pharmacokinetic parameters of geraniol and geranic acid in several tissues^a^.

		*C*_max_ (μg/g)	*T*_max_ (h)	*AUC* (μg/g × h)	*T*_1/2_ (h)
Brain	Geraniol^b^	—	—	—	—
	Geranic acid	1.38 ± 0.29	1.00	1.79 ± 0.37	n.c.
Kidney	Geraniol	—	—	—	—
	Geranic acid	86.57 ± 10.13	1.00	95.42 ± 9.83	n.c.
Liver	Geraniol	0.21 ± 0.04	1.00	1.58 ± 0.64	n.c.
	Geranic acid	6.31 ± 1.64	1.00	27.47 ± 2.86	n.c.
Lung	Geraniol	—	—	—	—
	Geranic acid	8.53 ± 1.29	1.00	27.45 ± 8.80	n.c.

*Note:* Data expressed as the mean ± SE.

Abbreviation: n.c., not calculated.

^a^ Parameters were calculated by analysis of the blood concentration versus time plots using the sparse sampling mode in the NCA module of the Phoenix WinNonlin Ver. 8.2.

^b^ Because geraniol was only detected in the brain of one animal, pharmacokinetic parameters for geraniol in the brain were not calculated.

## 4 Discussion

Geraniol has an oily form, extremely poor solubility, and a relatively high n‐octanol partition coefficient (Turina et al. 2006). Several formulations have been proposed to increase geraniol levels in the blood and tissues after administration (De Fazio et al. 2016; Pavan et al. 2018; Bonferoni et al. 2019; De Oliveira Junior et al. 2020). In the present study, we evaluated the pharmacokinetics profile of geraniol after oral administration in mice using a formulation mixed with corn oil because several pharmacological effects of geraniol have been investigated using this formulation (Shoff et al. 1991; Ozkaya et al. 2016; Huang et al. 2018). Geraniol did not degrade and remained stable in the corn oil formulation before oral administration. This report presents pharmacokinetic data on the blood and tissue concentrations after oral administration of geraniol in mice for the first time. Previously, it has been reported that the *C*_max_ of emulsified geraniol was relatively high (276 ± 15 μg/mL), and absolute bioavailability was also high (92%) in rats (Pavan et al. 2018). Furthermore, geraniol was detected in the cerebrospinal fluid after oral administration. However, despite a higher dose (200 mg/kg) in our study compared with the previous study (50 mg/kg) in rats, the *C*_max_ (0.05 ± 0.01 μg/mL) of geraniol was markedly lower in mice. In addition, we detected geraniol in the liver and brain, but not in the kidney and lungs, although geraniol was only detected in the brain of one animal. Conversely, the levels of geranic acid and the glucuronide conjugate of geraniol were markedly higher than the level of geraniol in the blood. Particularly, the *C*_max_ of geranic acid was the highest in the blood (14.97 ± 6.48 μg/mL), which was 300‐fold higher than that of geraniol in the blood. These results indicated that geraniol rapidly metabolized to geranic acid. The lower blood concentration of geraniol after oral administration may not only be due to differences in the fraction absorbed (*F*_a_) between formulations but also differences in intestinal availability (*F*_g_) and hepatic availability (*F*_h_) between mice and rats.

Geranic acid was the most abundant metabolite and was rapidly and widely distributed in the kidney, liver, lungs, and brain after the oral administration of geraniol. Notably, geranic acid and its conjugated metabolite were detected in the brain, and concentrations of geranic acid were higher than those of geraniol. Regarding the pharmacological effects of geranic acid, it has been reported that geranic acid shows antifungal, but not antibacterial, activity against *Trichophyton* sp. (Nagaki et al. 2011). Recently, Valdes, Calzada, and Mendieta‐Wejebe (2019) have reported that geranic acid shows antihyperglycemic activity in streptozotocin‐induced diabetic mice. In addition, geranic acid may act as a skin depigmenting agent by inhibiting the activity and expression of tyrosinase within melanocytes (Choi 2012). Also, Masuda et al. (2008) have been reported that geranic acid is the tyrosinase inhibitor because the *IC*_50_ value for tyrosinase of trans‐ and cis‐geranic acid was 0.14 and 2.3 mM, respectively. Recently, it has been found that tyrosinase inhibitors may be effective in Parkinson's disease (Li et al. 2022), indicating that the tyrosinase inhibitory activity of geranic acid may also be effective in animal models of Parkinson's disease (Rekha, Selvakumar, Santha, et al. 2013; Rekha, Selvakumar, Sethupathy, et al. 2013; Rekha and Selvakumar 2014). These results suggest that geranic acid, but not geraniol, plays an important physiological role in mice. Thus, geraniol is metabolized to geranic acid, and this metabolite is thought to exhibit several pharmacological effects in mice. To clarify this point, further studies are necessary to investigate several biological activities (anti‐inflammatory, antioxidant, or neuroprotective) of geranic acid.

We found geranic acid and a glucuronide conjugate of geraniol in plasma by LC–MS/MS and chemical hydrolysis after oral administration of geraniol in mice. Additionally, conjugated metabolites of geraniol, geranic acid, and 8‐hydroxygeraniol were detected in several tissues (data not shown). In human studies, geranic acid and 8‐carboxygeraniol were only detected in hydrolyzed urine, indicating that geranic acid and 8‐carboxygeraniol were metabolized to a conjugate of glucuronide and/or sulfate (Jäger et al. 2020). The conjugates of metabolites have been reported to play important physiological roles in vivo. For example, the major metabolites of quercetin are quercetin‐3‐glucuronide (Q3GA), quercetin‐3′‐sulfate (Q3'S), and isorhamnetin‐3‐glucuronide (I3GA) in human plasma (Manach et al. 1998; Moon et al. 2000; Mullen, Edwards, and Crozier 2006). When Q3GA and I3GA were administered to conscious spontaneously hypertensive rats, the mean blood pressure gradually decreased, and this hypotensive effect of Q3GA was abolished by specific inhibitors (Galindo et al. 2012). Similarly, resveratrol exhibited low bioavailability and rapidly metabolized to glucuronide–sulfate complexes such as resveratrol‐3‐*O*‐sulfate, resveratrol‐3‐*O*‐glucuronide, and resveratrol‐4′‐*O*‐glucuronide (Boocock, Faust, et al. 2007; Boocock, Patel, et al. 2007; Patel et al. 2010, 2011). Patel et al. (2013) have reported that resveratrol is delivered to target tissues in the form of a stable sulfate conjugate and shows potential beneficial effects in vivo, and the parent compound is gradually regenerated in a selected cell. These results suggest that orally administrated geraniol is metabolized to conjugates, either directly or after metabolizing to geranic acid and/or 8‐hydroxygeraiol, and the conjugates exhibit physiological effects in mice.

It has been reported that geranic acid, 3‐hydroxycitronellic acid, 8‐hydroxygeraniol, 8‐carboxygeraniol, and Hildebrandt acid were detected in the urine of rats after oral administration of geraniol. However, 8‐hydroxygeraniol was not detected in the present study. Similarly, 8‐hydroxygeraniol has not been detected in human urine (Jäger et al. 2020). These results suggest that 8‐hydroxygeraniol is rapidly metabolized to other metabolites or rapidly excreted from the body in mice and humans. Therefore, further studies are required to investigate the presence of 8‐carboxygeraniol and Hildebrandt acid in blood and urine after oral administration of geraniol to mice and to identify the primary metabolites.

In conclusion, geranic acid was mainly present in the form of metabolites in mice treated with geraniol, suggesting that various pharmacological effects of geraniol are induced by its metabolites.

## Author Contributions

**Yoshiaki Moriki:** conceptualization (equal); data curation (equal); writing – original draft (equal). **Ryo Mitsugi:** investigation (equal); methodology (equal). **Tomoyoshi Kayou:** formal analysis (equal); investigation (equal); methodology (equal). **Jumpei Horikoshi:** investigation (equal). **Yoshimasa Yamaguchi:** supervision (equal); writing – review and editing (equal). **Shuichi Shibuya:** methodology (equal); writing – review and editing (equal). **Takahiko Shimizu:** conceptualization (equal); writing – review and editing (equal).

## Conflicts of Interest

The authors declare no conflicts of interest.

## Data Availability

The data that support the findings of this study are available on request from the corresponding author.

## References

[fsn34653-bib-0001] Bonferoni, M. C. , L. Ferraro , B. Pavan , et al. 2019. “Uptake in the Central Nervous System of Geraniol Oil Encapsulated in Chitosan Oleate Following Nasal and Oral Administration.” Pharmaceutics 11: 106. 10.3390/pharmaceutics11030106. 30832389 PMC6471858

[fsn34653-bib-0002] Boocock, D. J. , G. E. Faust , K. R. Patel , et al. 2007. “Phase I Dose Escalation Pharmacokinetic Study in Healthy Volunteers of Resveratrol, a Potential Cancer Chemopreventive Agent.” Cancer Epidemiology, Biomarkers & Prevention 16: 1246–1252. 10.1158/1055-9965.epi-07-0022. 17548692

[fsn34653-bib-0003] Boocock, D. J. , K. R. Patel , G. E. Faust , et al. 2007. “Quantitation of Trans‐Resveratrol and Detection of Its Metabolites in Human Plasma and Urine by High Performance Liquid Chromatography.” Journal of Chromatography. B, Analytical Technologies in the Biomedical and Life Sciences 848: 182–187. 10.1016/j.jchromb.2006.10.017. 17097357 PMC2001235

[fsn34653-bib-0004] Chadha, A. , and K. M. Madyastha . 1984. “Metabolism of Geraniol and Linalool in the Rat and Effects on Liver and Lung Microsomal Enzymes.” Xenobiotica 14: 365–374. 10.3109/00498258409151425. 6475100

[fsn34653-bib-0005] Cho, M. , I. So , J. N. Chun , and J. H. Jeon . 2016. “The Antitumor Effects of Geraniol: Modulation of Cancer Hallmark Pathways (Review).” International Journal of Oncology 48: 1772–1782. 10.3892/ijo.2016.3427. 26983575 PMC4809657

[fsn34653-bib-0006] Choi, S. Y. 2012. “Inhibitory Effects of Geranic Acid Derivatives on Melanin Biosynthesis.” Journal of Cosmetic Science 63: 351–358. 23286867

[fsn34653-bib-0007] De Fazio, L. , E. Spisni , E. Cavazza , et al. 2016. “Dietary Geraniol by Oral or Enema Administration Strongly Reduces Dysbiosis and Systemic Inflammation in Dextran Sulfate Sodium‐Treated Mice.” Frontiers in Pharmacology 7: 38. 10.3389/fphar.2016.00038. 26973525 PMC4776160

[fsn34653-bib-0008] De Oliveira Junior, E. R. , E. Truzzi , L. Ferraro , et al. 2020. “Nasal Administration of Nanoencapsulated Geraniol/Ursodeoxycholic Acid Conjugate: Towards a New Approach for the Management of Parkinson's Disease.” Journal of Controlled Release 321: 540–552. 10.1016/j.jconrel.2020.02.033. 32092370

[fsn34653-bib-0009] El‐Emam, S. Z. , A. A. Soubh , A. K. Al‐Mokaddem , and D. M. Abo El‐Ella . 2020. “Geraniol Activates Nrf‐2/HO‐1 Signaling Pathway Mediating Protection Against Oxidative Stress‐Induced Apoptosis in Hepatic Ischemia‐Reperfusion Injury.” Naunyn‐Schmiedeberg's Archives of Pharmacology 393: 1849–1858. 10.1007/s00210-020-01887-1. 32417955

[fsn34653-bib-0010] Galindo, P. , I. Rodriguez‐Gómez , S. González‐Manzano , et al. 2012. “Glucuronidated Quercetin Lowers Blood Pressure in Spontaneously Hypertensive Rats via Deconjugation.” PLoS One 7: e32673. 10.1371/journal.pone.0032673. 22427863 PMC3299686

[fsn34653-bib-0011] Galle, M. , R. Crespo , B. R. Kladniew , S. M. Villegas , M. Polo , and M. G. de Bravo . 2014. “Suppression by Geraniol of the Growth of A549 Human Lung Adenocarcinoma Cells and Inhibition of the Mevalonate Pathway in Culture and In Vivo: Potential Use in Cancer Chemotherapy.” Nutrition and Cancer 66: 888–895. 10.1080/01635581.2014.916320. 24875281

[fsn34653-bib-0012] Huang, Y. , X. L. Yang , Y. H. Ni , and Z. M. Xu . 2018. “Geraniol Suppresses Proinflammatory Mediators in Phorbol 12‐Myristate 13‐Acetate With A23187‐Induced HMC‐1 Cells.” Drug Design, Development and Therapy 12: 2897–2903. 10.2147/dddt.s145702. 30254419 PMC6141105

[fsn34653-bib-0013] Jäger, T. , S. Bäcker , T. Brodbeck , E. Leibold , and M. Bader . 2020. “Quantitative Determination of Urinary Metabolites of Geraniol by Ultra‐Performance Liquid Chromatography‐Tandem Mass Spectrometry (UPLC‐MS/MS).” Analytical Methods 12: 5718–5728. 10.1039/d0ay01582b. 33220670

[fsn34653-bib-0014] Khan, A. Q. , R. Khan , W. Qamar , et al. 2013. “Geraniol Attenuates 12‐O‐Tetradecanoylphorbol‐13‐Acetate (TPA)‐induced Oxidative Stress and Inflammation in Mouse Skin: Possible Role of p38 MAP Kinase and NF‐κB.” Experimental and Molecular Pathology 94: 419–429. 10.1016/j.yexmp.2013.01.006. 23399806

[fsn34653-bib-0015] Lapczynski, A. , S. P. Bhatia , R. J. Foxenberg , C. S. Letizia , and A. M. Api . 2008. “Fragrance Material Review on Geraniol.” Food and Chemical Toxicology 46: S160–S170. 10.1016/j.fct.2008.06.048. 18640215

[fsn34653-bib-0037] Li, Q. , J. Mo , B. Xiong , et al. 2022. “Discovery of Resorcinol‐Based Polycyclic Structures as Tyrosinase Inhibitors for Treatment of Parkinson’s Disease..” ACS Chemical Neuroscience 13, no. 1: 81–96. 10.1021/acschemneuro.1c00560. 34882402

[fsn34653-bib-0016] Manach, C. , C. Morand , V. Crespy , et al. 1998. “Quercetin Is Recovered in Human Plasma as Conjugated Derivatives Which Retain Antioxidant Properties.” FEBS Letters 426: 331–336. 10.1016/s0014-5793(98)00367-6. 9600261

[fsn34653-bib-0017] Marcuzzi, A. , S. Crovella , and A. Pontillo . 2011. “Geraniol Rescues Inflammation in Cellular and Animal Models of Mevalonate Kinase Deficiency.” In Vivo 25: 87–92. 21282739

[fsn34653-bib-0018] Masuda, T. , Y. Odaka , N. Ogawa , K. Nakamoto , and H. Kuninaga . 2008. “Identification of Geranic Acid, a Tyrosinase Inhibitor in Lemongrass ( *Cymbopogon citratus* ).” Journal of Agricultural and Food Chemistry 56: 597–601. 10.1021/jf072893l. 18081247

[fsn34653-bib-0019] Moon, J. H. , R. Nakata , S. Oshima , T. Inakuma , and J. Terao . 2000. “Accumulation of Quercetin Conjugates in Blood Plasma After the Short‐Term Ingestion of Onion by Women.” American Journal of Physiology, Regulatory, Integrative and Comparative Physiology 279: R461–R467. 10.1152/ajpregu.2000.279.2.r461. 10938233

[fsn34653-bib-0020] Mullen, W. , C. A. Edwards , and A. Crozier . 2006. “Absorption, Excretion and Metabolite Profiling of Methyl‐, Glucuronyl‐, Glucosyl‐ and Sulpho‐Conjugates of Quercetin in Human Plasma and Urine After Ingestion of Onions.” British Journal of Nutrition 96: 107–116. 10.1079/bjn20061809. 16869998

[fsn34653-bib-0021] Nagaki, M. , T. Narita , H. Ichikawa , J. Kawakami , and A. Nakane . 2011. “Antibacterial and Antifungal Activities of Isoprenoids.” Transactions of the Materials Research Society of Japan 36: 55–58. 10.14723/tmrsj.36.55.

[fsn34653-bib-0022] Ozkaya, A. , Z. Sahin , A. O. Gorgulu , A. Yuce , and S. Celik . 2016. “Geraniol Attenuates Hydrogen Peroxide‐Induced Liver Fatty Acid Alterations in Male Rats.” Journal of Intercultural Ethnopharmacology 6: 29–35. 10.5455/jice.20160928012410. 28163957 PMC5289085

[fsn34653-bib-0023] Patel, K. R. , C. Andreadi , R. G. Britton , et al. 2013. “Sulfate Metabolites Provide an Intracellular Pool for Resveratrol Generation and Induce Autophagy With Senescence.” Science Translational Medicine 5: 205ra133. 10.1126/scitranslmed.3005870. 24089405

[fsn34653-bib-0024] Patel, K. R. , V. A. Brown , D. J. Jones , et al. 2010. “Clinical Pharmacology of Resveratrol and Its Metabolites in Colorectal Cancer Patients.” Cancer Research 70: 7392–7399. 10.1158/0008-5472.can-10-2027. 20841478 PMC2948608

[fsn34653-bib-0025] Patel, K. R. , E. Scott , V. A. Brown , A. J. Gescher , W. P. Steward , and K. Brown . 2011. “Clinical Trials of Resveratrol.” Annals of the New York Academy of Sciences 1215: 161–169. 10.1111/j.1749-6632.2010.05853.x. 21261655

[fsn34653-bib-0026] Pavan, B. , A. Dalpiaz , L. Marani , et al. 2018. “Geraniol Pharmacokinetics, Bioavailability and Its Multiple Effects on the Liver Antioxidant and Xenobiotic‐Metabolizing Enzymes.” Frontiers in Pharmacology 9: 18. 10.3389/fphar.2018.00018. 29422862 PMC5788896

[fsn34653-bib-0027] Rekha, K. R. , and G. P. Selvakumar . 2014. “Gene Expression Regulation of Bcl2, Bax and Cytochrome‐C by Geraniol on Chronic MPTP/Probenecid Induced C57BL/6 Mice Model of Parkinson's Disease.” Chemico‐Biological Interactions 217: 57–66. 10.1016/j.cbi.2014.04.010. 24768735

[fsn34653-bib-0028] Rekha, K. R. , G. P. Selvakumar , K. Santha , and R. I. Sivakamasundari . 2013. “Geraniol Attenuates α‐Synuclein Expression and Neuromuscular Impairment Through Increase Dopamine Content in MPTP Intoxicated Mice by Dose Dependent Manner.” Biochemical and Biophysical Research Communications 440: 664–670. 10.1016/j.bbrc.2013.09.122. 24103762

[fsn34653-bib-0029] Rekha, K. R. , G. P. Selvakumar , S. Sethupathy , K. Santha , and R. I. Sivakamasundari . 2013. “Geraniol Ameliorates the Motor Behavior and Neurotrophic Factors Inadequacy in MPTP‐Induced Mice Model of Parkinson's Disease.” Journal of Molecular Neuroscience 51: 851–862. 10.1007/s12031-013-0074-9. 23943375 PMC3824202

[fsn34653-bib-0030] Shoff, S. M. , M. Grummer , M. B. Yatvin , and C. E. Elson . 1991. “Concentration‐Dependent Increase of Murine P388 and B16 Population Doubling Time by the Acyclic Monoterpene Geraniol.” Cancer Research 51: 37–42. 1988098

[fsn34653-bib-0031] Takechi, H. , and M. Miyazawa . 2006. “Biotransformation of Geraniol by the Larvae of Common Cutworm ( *Spodoptera litura* ).” Journal of Oleo Science 55: 143–149. 10.5650/jos.62.313.

[fsn34653-bib-0032] Thapa, D. , R. Losa , B. Zweifel , and R. J. Wallace . 2012. “Sensitivity of Pathogenic and Commensal Bacteria From the Human Colon to Essential Oils.” Microbiology 158: 2870–2877. 10.1099/mic.0.061127-0. 22878397

[fsn34653-bib-0033] Turina, A. V. , M. V. Nolan , J. A. Zygadlo , and M. A. Perillo . 2006. “Natural Terpenes: Self‐Assembly and Membrane Partitioning.” Biophysical Chemistry 122: 101–113. 10.1016/j.bpc.2006.02.007. 16563603

[fsn34653-bib-0034] Valdes, M. , F. Calzada , and J. Mendieta‐Wejebe . 2019. “Structure‐Activity Relationship Study of Acyclic Terpenes in Blood Glucose Levels: Potential α‐Glucosidase and Sodium Glucose Cotransporter (SGLT‐1) Inhibitors.” Molecules 24: 4020. 10.3390/molecules24224020. 31698833 PMC6891574

[fsn34653-bib-0035] Wang, J. , B. Su , H. Zhu , C. Chen , and G. Zhao . 2016. “Protective Effect of Geraniol Inhibits Inflammatory Response, Oxidative Stress and Apoptosis in Traumatic Injury of the Spinal Cord Through Modulation of NF‐κB and p38 MAPK.” Experimental and Therapeutic Medicine 12: 3607–3613. 10.3892/etm.2016.3850. 28105094 PMC5228434

[fsn34653-bib-0036] Wittig, C. , C. Scheuer , J. Parakenings , M. D. Menger , and M. W. Laschke . 2015. “Geraniol Suppresses Angiogenesis by Downregulating Vascular Endothelial Growth Factor (VEGF)/VEGFR‐2 Signaling.” PLoS One 10: e0131946. 10.1371/journal.pone.0131946. 26154255 PMC4496091

